# Announcements

**Published:** 2015-05-01

## Drinking Water Week — May 3–9, 2015

The United States has one of the safest public drinking water supplies in the world (1). Tap water not only provides water for daily personal activities such as drinking, bathing, and cooking, but also benefits communities by providing water to serve businesses, schools, and hospitals. A safe supply of water is an important part of overall health. May 3–9, 2015, is Drinking Water Week, an annual observance. This year’s theme “What Do You Know About H_2_O?” underscores the many ways consumers can learn more about their water (2).

Disinfection and treatment practices, as well as the environmental regulation of water pollutants, have substantially improved domestic water quality during the past century and have led to a marked decrease in the incidence of waterborne diseases such as typhoid fever (3–5). Despite these improvements, sources of drinking water still can become contaminated and lead to adverse health effects (6).

New challenges to the U.S. water supply include deteriorating drinking water infrastructure, the impact of climate change on water availability and quality, chemical contamination of water sources, emerging pathogens, and the development of new ways to obtain and use water.

Drinking Water Week is a time to highlight the importance that providing safe drinking water and protecting and reinvesting in water infrastructure has to U.S. public health.

## References

[1] US Environmental Protection Agency (2009). Drinking water and health: what you need to know.

[2] American Water Works Association (2015). Drinking Water Week 2015.

[3] CDC (1999). Safer and healthier foods. MMWR Morb Mortal Wkly Rep.

[4] CDC (2012). Summary of notifiable diseases—United States, 2010. MMWR Morb Mortal Wkly Rep.

[5] Cutler D, Miller G (2004). The role of public health improvements in health advances: the 20th century United States.

[6] US Environmental Protection Agency (2011). Drinking water contaminants.

